# Supplementary material for: Identification of the Intestinal Microbes Associated with Locomotion
Source: Int J Mol Sci. 2023 Jul 13;24(14):11392. doi: 10.3390/ijms241411392 (PMC10380270; doi:10.3390/ijms241411392)
Supplement: Supplementary file 1 [file ijms-24-11392-s001.zip › ijms-2465882-supplementary.pdf]

Supplementary Materials

Supplemental Figures

Figure S1

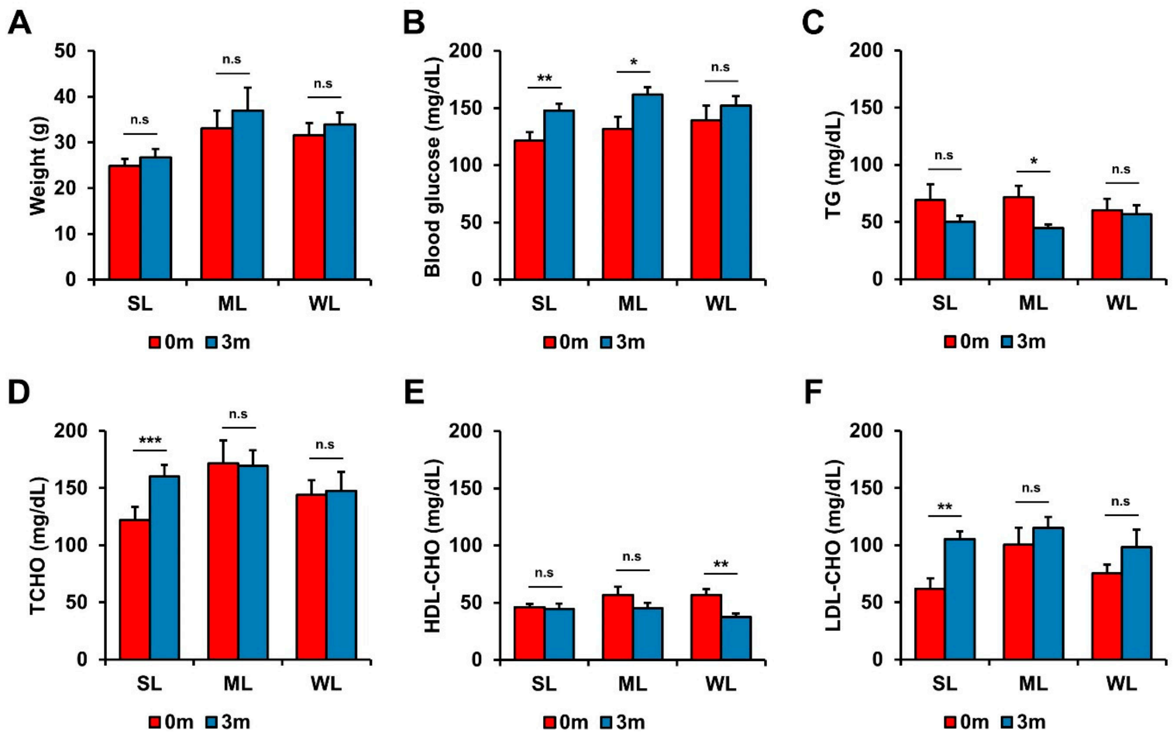

Figure S1. The effects of FMT on the changes in the blood glucose, blood pressure, and lipid levels. The average values of **(A)** Weight, **(B)** Blood glucose, **(C)** Total glycerol (TG), **(D)** Total cholesterol (TCHO), **(E)** HDL-cholesterol (HDL-CHO) **(F)** LDL-cholesterol (LDL-CHO) of each group are shown. The values are represented as mean ± SEM. 0m represents before the gut microbiome replacement, and 3m represents 3 months after the gut microbiome replacement. *$p < 0.05$; **$p < 0.01$; ***$p < 0.001$; *n.s*: not significant ($p > 0.05$)

Figure S2

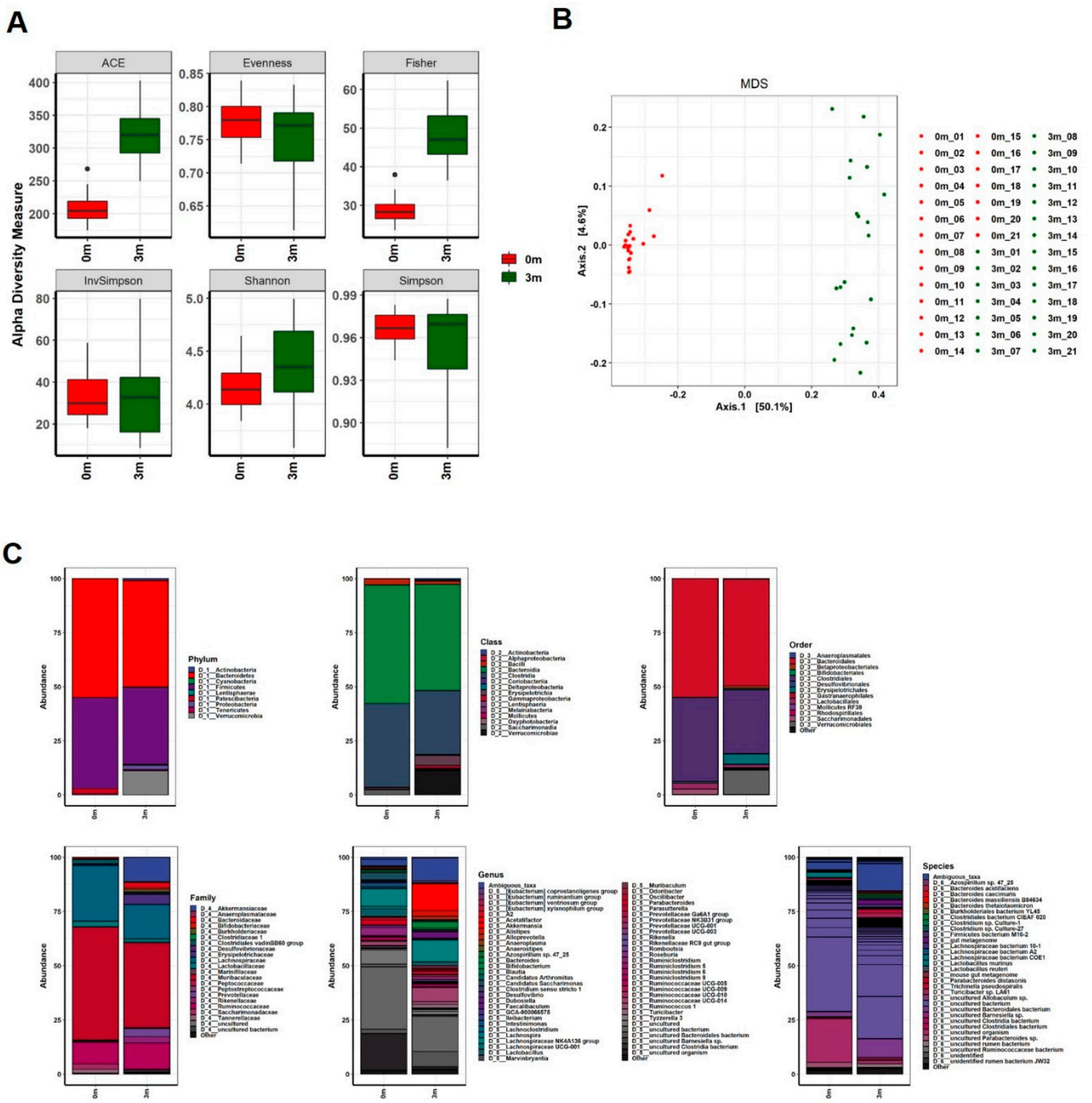

Figure S2. Changes in the gut microbiome before and after FMT. (A) α-diversity, **(B)** β-diversity and **(C)** Abundance bar plot are shown that the mice gut microbiome were effectively exchanged by gut microbiome replacement.

Figure S3

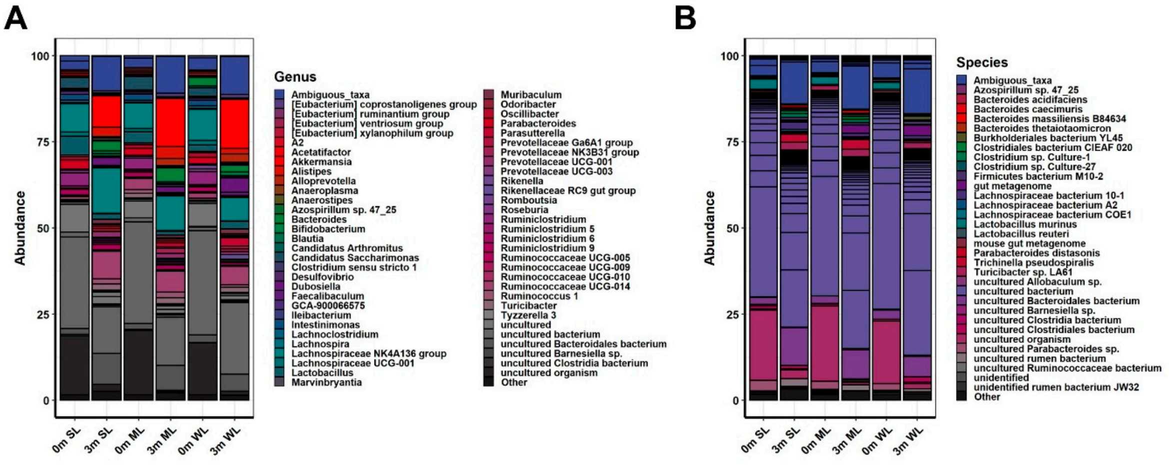

Figure S3. The composition of the gut microbiome in experimental mice before and after FMT. The relative changes in the gut microbiome composition were analyzed at the **(A)** genus and **(B)** species levels. The average abundance values of each group were calculated to obtain the abundance values.

Figure S4

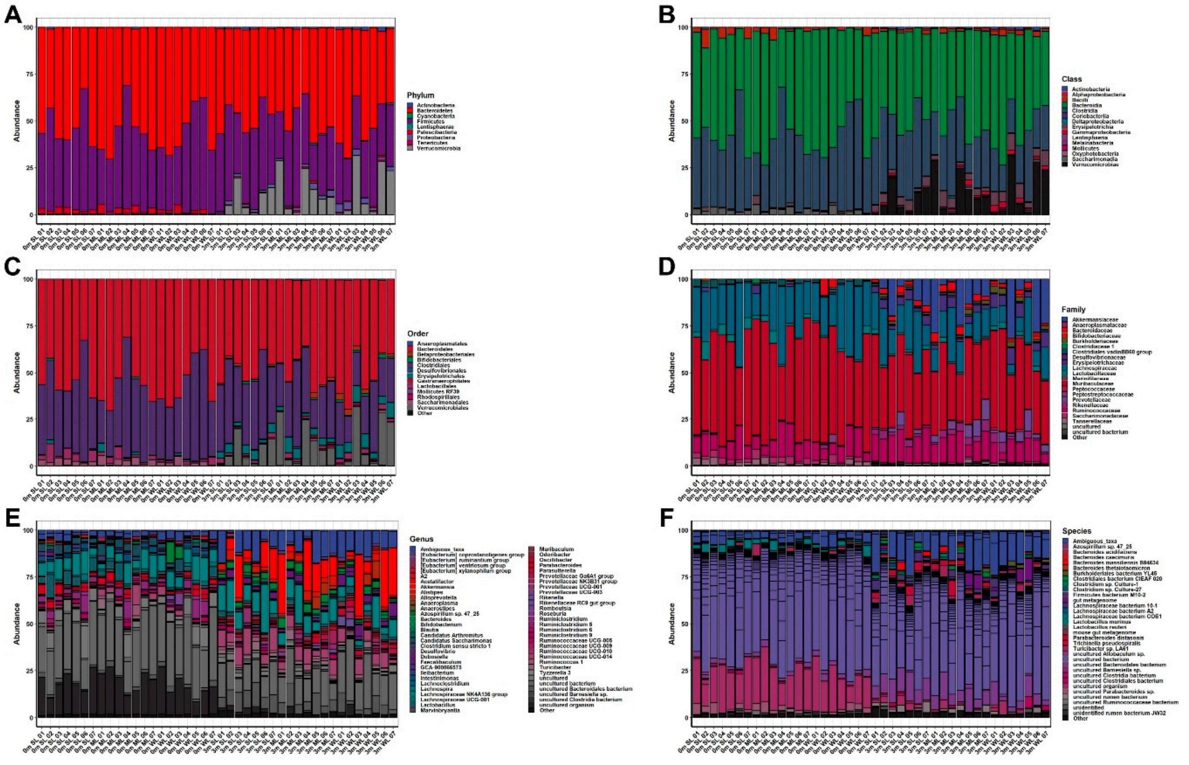

Figure S4. Changes in the composition of the gut microbiome of each mouse before and after the gut microbiome replacement. (A) Phylum level, **(B)** Class level, **(C)** Order level, **(D)** Family level, **(E)** Genus level, and **(F)** Species level are shown.

Figure S5

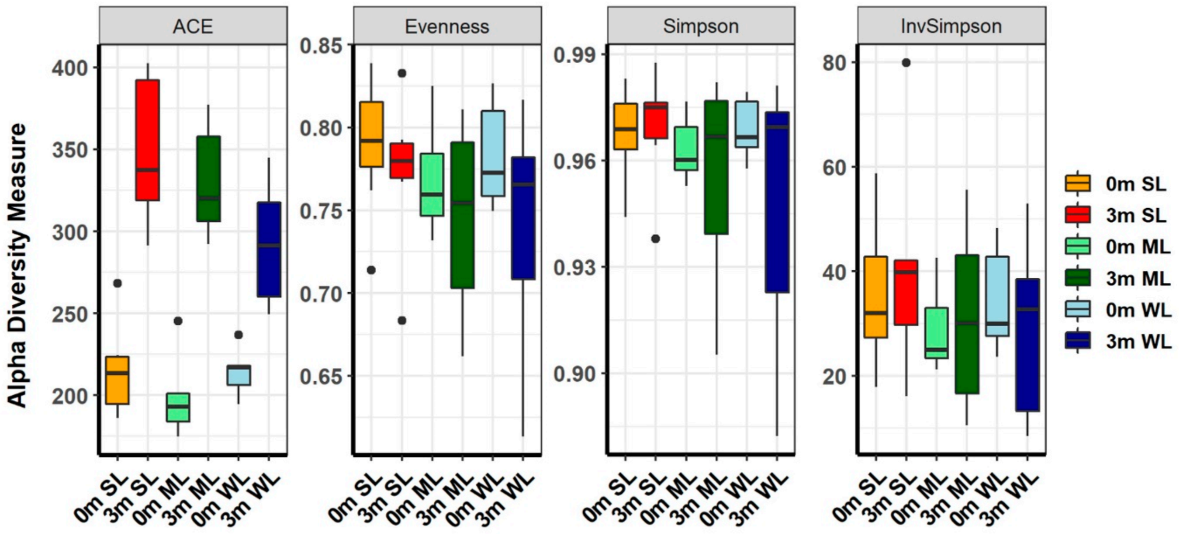

Figure S5. The α-diversity indices of the gut microbiome of the SL, ML, and WL groups. The species richness and diversity, as calculated by ACE richness, Evenness, Simpson, and InvSimpson, are shown for before (0m) and after (3m) FMT for each of the SL, ML, and WL groups.

Table S1. The valid reads of 16S rRNA amplicon sequence

Group	0m SL	0m ML	0m WL	3m SL	3m ML	3m WL
Total reads	41,229 ± 1,453	39,046 ± 1,681	42,761 ± 1,174	38,942 ± 1,839	39,860 ± 1,014	34,185 ± 3,168

All values are the mean ± SEM. OTUs, operational taxonomic units

Table S2. Comparison of taxonomy abundance at the phylum level

Phylum	0m SL	0m ML	0m WL	3m SL	3m ML	3m WL	0m	3m
**Verrucomicrobia**	0	0	0	7.853	12.561	13.258	0	11.224
**Bacteroidetes**	52.614	56.302	55.709	47.460	49.487	50.848	54.875	49.265
**Firmicutes**	44.348	40.159	41.932	41.797	34.258	30.733	42.146	35.596
**Patescibacteria**	2.483	2.917	1.831	0.204	0.460	0.406	2.410	0.357
**Actinobacteria**	0.204	0.185	0.039	0.809	0.561	1.158	0.143	0.842
**Proteobacteria**	0.328	0.363	0.329	1.332	1.790	2.798	0.340	1.973
**Tenericutes**	0.023	0.072	0.160	0.464	0.546	0.492	0.085	0.501
**Cyanobacteria**	0.002	0.002	0	0.079	0.335	0.307	0.001	0.240
**Lentisphaerae**	0	0	0	0	0.002	0	0	0.001

Table S3. Comparison of taxonomy abundance at the class level

Class	0m SL	0m ML	0m WL	3m SL	3m ML	3m WL	0m	3m
**Verrucomicrobiae**	0	0	0	7.853	12.561	13.259	0	11.224
**Bacteroidia**	52.614	56.302	55.709	47.460	49.487	50.849	54.875	49.265
**Clostridia**	39.625	36.717	40.097	36.701	29.373	22.466	38.813	29.513
**Erysipelotrichia**	0.483	1.032	0.412	3.791	3.818	6.275	0.643	4.628
**Saccharimonadia**	2.483	2.917	1.831	0.204	0.460	0.406	2.410	0.357
**Bacilli**	4.240	2.410	1.422	1.305	1.067	1.991	2.691	1.454
**Actinobacteria**	0.143	0.132	0	0.642	0.404	0.972	0.092	0.673
**Coriobacteriia**	0.061	0.053	0.039	0.167	0.157	0.186	0.051	0.170
**Deltaproteobacteria**	0.175	0.160	0.187	0.320	0.299	0.163	0.174	0.260
**Gammaproteobacteria**	0.097	0.137	0.100	0.635	1.033	2.118	0.111	1.262
**Mollicutes**	0.023	0.072	0.160	0.464	0.546	0.492	0.085	0.501
**Alphaproteobacteria**	0.056	0.066	0.041	0.378	0.458	0.517	0.055	0.451
**Melainabacteria**	0	0	0	0.078	0.331	0.307	0	0.239
**Oxyphotobacteria**	0.002	0.002	0	0.001	0.004	0	0.001	0.002
**Lentisphaeria**	0	0	0	0	0.002	0	0	0.001

Table S4. Comparison of taxonomy abundance at the order level

Order	0m SL	0m ML	0m WL	3m SL	3m ML	3m WL	0m	3m
**Verrucomicrobiales**	0	0	0	7.853	12.561	13.259	0	11.224
**Bacteroidales**	52.614	56.301	55.709	47.408	49.432	50.840	54.875	49.227
**Clostridiales**	39.626	36.717	40.097	36.701	29.373	22.466	38.813	29.513
**Erysipelotrichales**	0.483	1.032	0.412	3.791	3.818	6.275	0.643	4.628
**Saccharimonadales**	2.483	2.917	1.831	0.204	0.460	0.406	2.410	0.357
**Lactobacillales**	4.239	2.410	1.422	1.305	1.060	1.991	2.691	1.452
**Bifidobacteriales**	0.143	0.132	0	0.642	0.404	0.972	0.092	0.673
**Desulfovibrionales**	0.167	0.160	0.187	0.320	0.299	0.163	0.171	0.260
**Betaproteobacteriales**	0.004	0.004	0.022	0.635	1.031	2.115	0.010	1.260
**Anaeroplasmatales**	0.003	0	0	0.177	0.260	0.328	0.001	0.255
**Mollicutes RF39**	0.019	0.072	0.160	0.238	0.285	0.162	0.084	0.228
**Rhodospirillales**	0	0	0	0.372	0.443	0.515	0	0.444
**Gastranaerophilales**	0	0	0	0.078	0.331	0.307	0	0.239
**Other < 0.5%**	0.219	0.256	0.158	0.276	0.244	0.201	0.211	0.240

Table S5. Comparison of taxonomy abundance at the family level

Family	0m SL	0m ML	0m WL	3m SL	3m ML	3m WL	0m	3m
**Akkermansiaceae**	0	0	0	7.859	12.572	13.263	0	11.231
**Muribaculaceae**	50.047	54.492	51.805	39.713	36.466	41.233	52.115	39.137
**Lachnospiraceae**	26.267	23.014	27.684	21.051	15.364	11.465	25.655	15.960
**Erysipelotrichaceae**	0.483	1.033	0.412	3.793	3.822	6.277	0.643	4.631
**Saccharimonadaceae**	2.483	2.919	1.831	0.204	0.461	0.406	2.411	0.357
**Tannerellaceae**	2.131	1.557	1.398	0.542	0.575	0.334	1.695	0.484
**Lactobacillaceae**	4.219	2.393	1.402	1.304	1.048	1.984	2.671	1.445
**Ruminococcaceae**	9.911	11.034	9.571	14.291	12.639	9.674	10.172	12.201
**Prevotellaceae**	0	0	0.095	1.770	4.964	4.480	0.032	3.738
**Bacteroidaceae**	0	0	1.901	2.496	3.535	1.773	0.634	2.601
**Clostridiales vadinBB60 group**	2.630	1.692	1.452	0.605	0.840	0.580	1.925	0.675
**Rikenellaceae**	0.444	0.274	0.516	2.555	3.539	2.627	0.411	2.907
**Bifidobacteriaceae**	0.143	0.132	0	0.642	0.404	0.972	0.092	0.673
**Desulfovibrionaceae**	0.167	0.160	0.187	0.320	0.299	0.163	0.171	0.260
**Peptococcaceae**	0.192	0.174	0.199	0.345	0.307	0.492	0.188	0.381
**Burkholderiaceae**	0.004	0.004	0.022	0.635	1.033	2.116	0.010	1.261
**Clostridiaceae 1**	0.237	0.461	0.243	0.148	0.101	0.102	0.314	0.117
**Anaeroplasmataceae**	0.003	0	0	0.177	0.260	0.328	0.001	0.255
**Peptostreptococcaceae**	0.216	0.156	0.788	0.017	0.005	0.025	0.386	0.016
**Marinifilaceae**	0	0	0	0.343	0.385	0.401	0	0.376
**uncultured bacterium**	0.009	0.022	0.151	0.167	0.153	0.063	0.061	0.128
**uncultured**	0	0	0	0.373	0.444	0.516	0	0.444
**uncultured bacterium**	0	0	0	0.045	0.291	0.249	0	0.195
**Other < 0.5%**	0.414	0.483	0.344	0.607	0.494	0.477	0.413	0.526

Table S6. Comparison of taxonomy abundance at the genus level

Genus	0m SL	0m ML	0m WL	3m SL	3m ML	3m WL	0m	3m
Akkermansia	0	0	0	9.032	13.995	14.186	0	12.404
Ambiguous_taxa	2.492	2.677	3.578	10.001	10.649	10.936	2.916	10.528
uncultured organism	17.178	18.714	15.065	2.028	0.572	1.177	16.985	1.259
uncultured bacterium	26.627	29.428	30.275	13.581	14.046	20.747	28.777	16.124
Turicibacter	0.233	1.225	0.469	1.819	1.779	1.443	0.642	1.680
uncultured Bacteroidales bacterium	1.637	1.801	2.220	8.953	7.195	4.957	1.886	7.035
Lachnospiraceae NK4A136 group	8.224	7.421	9.021	13.070	10.054	6.824	8.222	9.982
[Eubacterium] coprostanoligenes group	0.062	0.037	0.027	0.627	1.073	1.033	0.042	0.911
Candidatus Saccharimonas	3.168	3.918	2.464	0.240	0.519	0.452	3.184	0.404
Parabacteroides	2.713	2.070	1.852	0.643	0.642	0.375	2.211	0.553
Lactobacillus	5.346	3.256	1.855	1.550	1.183	2.167	3.486	1.633
Ruminococcaceae UCG-014	0.856	3.229	1.520	7.999	5.978	5.385	1.869	6.454
Ruminiclostridium	3.877	3.216	3.818	0.567	0.524	0.414	3.637	0.502
Alloprevotella	0	0	0.067	0.238	2.037	2.484	0.022	1.586
Bacteroides	0	0	2.519	2.918	3.966	1.962	0.840	2.949
uncultured bacterium	1.338	1.197	1.007	0.391	0.540	0.263	1.181	0.398
Alistipes	0.566	0.370	0.359	2.764	3.405	1.563	0.432	2.577
Faecalibaculum	0.306	0	0.002	0.179	0.338	0.988	0.103	0.502
Prevotellaceae UCG-001	0	0	0.055	1.724	1.387	0.875	0.018	1.329
Muribaculum	0.034	0	0.079	1.266	0.768	1.317	0.038	1.117
Bifidobacterium	0.180	0.188	0	0.757	0.451	1.052	0.123	0.753
Intestinimonas	1.664	1.852	1.553	0.113	0.208	0.194	1.690	0.172
Desulfovibrio	0.212	0.216	0.251	0.095	0.002	0.034	0.227	0.044
uncultured	8.100	4.857	6.725	2.180	1.097	1.301	6.561	1.526
[Eubacterium] ventriosum group	0.007	0.539	0.317	0.001	0.059	0.002	0.288	0.021
Dubosiella	0	0	0	2.240	1.806	4.149	0	2.732
Oscillibacter	0.963	1.079	1.338	0.468	0.459	0.411	1.127	0.446
Prevotellaceae NK3B31 group	0	0	0	0.060	1.456	0.776	0	0.764
[Eubacterium] ruminantium group	0	0	0	0.256	0.024	0.102	0	0.128
uncultured	0.663	0.492	0.526	1.241	1.035	0.315	0.560	0.864
Ambiguous_taxa	1.577	0.693	0.649	0.143	0.214	0.256	0.973	0.204
Blautia	0.302	0.385	0.491	0.676	0.276	0.107	0.392	0.353
Rikenellaceae RC9 gut group	0	0	0.320	0.079	0.454	1.356	0.107	0.629
uncultured	0	0	0	0.170	0.278	0.062	0	0.170
[Eubacterium] xylanophilum group	0.406	0.411	0.497	0.376	0.287	0.218	0.438	0.294
Lachnospiraceae UCG-001	1.260	0.921	1.264	0.214	0.146	0.069	1.149	0.143
uncultured	0.227	0.225	0.248	0.406	0.340	0.527	0.233	0.424
uncultured Clostridia bacterium	0.403	0.204	0.055	0.017	0.028	0.009	0.221	0.018
Marvinbryantia	0.502	0.516	0.353	0.051	0.032	0.143	0.457	0.075
Parasutterella	0	0	0.028	0.753	1.157	2.305	0.009	1.405
Ruminococcaceae UCG-009	0.356	0.342	0.289	0.202	0.168	0.113	0.329	0.161
Prevotellaceae Ga6A1 group	0	0	0	0.002	0.413	0.876	0	0.431
Ruminiclostridium 9	1.635	1.353	1.375	1.549	1.308	0.672	1.454	1.176
Ruminiclostridium 6	0.318	0.419	0.255	0.470	0.171	0.253	0.331	0.298
Clostridium sensu stricto 1	0.298	0.427	0.330	0.143	0.052	0.035	0.352	0.077
Roseburia	0.712	0.550	0.769	0.479	0.309	0.238	0.677	0.342
Lachnoclostridium	0.727	0.509	0.729	0.287	0.168	0.115	0.655	0.190
Candidatus Arthromitus	0	0.161	0	0.034	0.064	0.075	0.054	0.058
GCA-900066575	0.936	0.532	0.655	0.167	0.084	0.085	0.708	0.112
Ileibacterium	0	0	0	0.045	0.139	0.132	0	0.105
A2	0.484	0.432	0.620	0.070	0.030	0.143	0.512	0.081

**Ruminiclostridium 5**	0.597	0.466	0.429	0.193	0.173	0.090	0.497	0.152
**Anaeroplasma**	0.004	0	0	0.212	0.288	0.362	0.001	0.287
**Lachnospira**	0.305	0.246	0.325	0.007	0.003	0.004	0.292	0.005
**Romboutsia**	0.273	0.209	1.086	0.021	0.006	0.027	0.523	0.018
**Ruminococcus 1**	0	1.053	0.055	1.457	1.635	0.708	0.369	1.267
**Anaerostipes**	0	0	0	0.647	0.309	0.197	0	0.385
**Ruminococcaceae UCG-010**	0.065	0.046	0.037	0.225	0.272	0.243	0.049	0.247
**Tyzzerella 3**	0.309	0.287	0.161	0.030	0.018	0.010	0.252	0.019
**Odoribacter**	0	0	0	0.278	0.345	0.416	0	0.346
**uncultured bacterium**	0.011	0.030	0.200	0.195	0.173	0.069	0.080	0.146
**Acetatifactor**	0.240	0.116	0.160	0.171	0.106	0.052	0.172	0.110
**Azospirillum sp. 47_25**	0	0	0	0.205	0.154	0.238	0	0.199
**Prevotellaceae UCG-003**	0	0	0	0	0.289	0	0	0.096
**Rikenella**	0	0	0	0.221	0.112	0	0	0.111
**uncultured bacterium**	0	0	0	0.125	0.054	0.122	0	0.100
**uncultured bacterium**	0	0	0	0.052	0.331	0.268	0	0.217
**Ruminococcaceae UCG-005**	0.036	0.095	0.037	0.029	0.070	0.026	0.056	0.042
**uncultured Barnesiella sp.**	0	0	0	0.006	0.139	0	0	0.049
**Other < 0.5%**	1.570	1.589	1.624	2.597	2.163	1.492	1.594	2.084

Table S7. Comparison of taxonomy abundance at the species level

Species	0m SL	0m ML	0m WL	3m SL	3m ML	3m WL	0m	3m
uncultured bacterium	0	0	0	10.874	16.803	16.467	0	14.715
Ambiguous_taxa	2.968	3.144	4.351	12.159	12.520	12.949	3.488	12.543
uncultured organism	20.413	21.830	18.152	2.519	0.673	1.413	20.131	1.535
uncultured bacterium	31.830	34.678	36.404	16.599	16.672	24.641	34.304	19.304
Turicibacter sp. LA61	0.280	1.422	0.572	2.209	2.095	1.728	0.758	2.011
uncultured Bacteroidales bacterium	1.952	2.120	2.678	10.929	8.443	5.913	2.250	8.428
Trichinella pseudospiralis	0	0	0	2.412	2.794	0.385	0	1.864
Lachnospiraceae bacterium 10-1	0	0	0	2.084	1.042	0.976	0	1.367
uncultured bacterium	3.774	4.659	3.036	0.302	0.597	0.542	3.823	0.481
uncultured Parabacteroides sp.	3.049	2.378	1.958	0.052	0.008	0.006	2.462	0.022
Lactobacillus murinus	2.973	2.227	1.971	0.204	0.304	0.234	2.391	0.247
uncultured rumen bacterium	0.016	0.262	0	2.237	1.621	0.920	0.093	1.593
uncultured bacterium	4.754	3.764	4.535	0.670	0.615	0.487	4.351	0.591
gut metagenome	0	0	0.090	0.280	2.352	3.026	0.030	1.886
uncultured bacterium	1.604	1.419	1.212	0.470	0.636	0.310	1.412	0.472
uncultured bacterium	0.104	0	0.011	2.976	3.114	1.306	0.038	2.466
uncultured bacterium	4.402	4.013	4.406	5.498	4.915	2.200	4.274	4.205
uncultured bacterium	0.395	0	0.002	0.224	0.397	1.170	0.133	0.597
uncultured bacterium	0.044	0	0.103	1.552	0.926	1.557	0.049	1.345
uncultured bacterium	1.963	2.142	1.795	0.020	0.107	0.112	1.967	0.080
uncultured bacterium	0.256	0.252	0.296	0.120	0.003	0.039	0.268	0.054
Clostridiales bacterium CIEAF 020	0	0	0	0.814	0.765	0.236	0	0.605
Bacteroides caecimuris	0	0	0	0.604	0.707	0.204	0	0.505
Bacteroides acidifaciens	0	0	0	0.115	0.287	0.111	0	0.171
uncultured bacterium	0	0	0	1.914	1.352	4.042	0	2.436
uncultured bacterium	0.528	0.732	0.880	0.297	0.349	0.363	0.714	0.336
uncultured bacterium	0	0	0	0.071	1.742	0.946	0	0.920
uncultured bacterium	3.435	2.662	3.701	1.859	0.746	0.809	3.266	1.138
uncultured bacterium	0	0	0	0.310	0.029	0.122	0	0.154
Clostridium sp. Culture-1	0.253	0.227	0.201	1.128	0.987	0.208	0.227	0.774
Ambiguous_taxa	1.899	0.823	0.771	0.174	0.250	0.302	1.164	0.242
unidentified	0.174	0.322	0.506	0.323	0.067	0.419	0.334	0.270
uncultured Bacteroidales bacterium	0.213	0	0.250	0.138	0.419	0.342	0.154	0.300
uncultured Clostridiales bacterium	0.873	0.553	0.441	0.879	0.444	1.464	0.622	0.929
Ambiguous_taxa	0	0	0.428	0.076	0.522	1.621	0.143	0.740
Ambiguous_taxa	0	0	0	0.204	0.329	0.075	0	0.202
uncultured bacterium	0.490	0.489	0.607	0.449	0.347	0.265	0.529	0.354
uncultured bacterium	1.547	1.080	1.478	0.183	0.139	0.052	1.369	0.125
uncultured organism	0	0	0	0.285	0.266	0.485	0	0.345
unidentified	0.391	0.351	0.450	0.162	0.117	0.096	0.398	0.125
Parabacteroides distasonis	0	0	0	0.363	0.206	0.067	0	0.212
Lachnospiraceae bacterium COE1	0.246	0.270	0.387	0.635	0.189	0.146	0.301	0.323
uncultured Clostridia bacterium	0.487	0.235	0.065	0.021	0.034	0.010	0.262	0.022
uncultured bacterium	0.523	0.506	0.342	0.026	0.001	0.159	0.457	0.062
uncultured Allobaculum sp.	0	0	0	0	0.082	0	0	0.027
uncultured bacterium	0	0	0.037	0	0.509	0.752	0.012	0.420
Ambiguous_taxa	0.430	0.410	0.340	0.232	0.198	0.134	0.393	0.188
uncultured bacterium	0.275	0.265	0.297	0.205	0.138	0.141	0.279	0.161
Firmicutes bacterium M10-2	0	0	0	0.768	0.705	0.793	0	0.755
Ambiguous_taxa	0	0	0	0.001	0.499	1.006	0	0.502
uncultured bacterium	0.884	0.776	0.705	1.575	1.202	0.545	0.788	1.107

**uncultured bacterium**	0.173	0.242	0.237	1.943	1.479	0.706	0.217	1.376
**uncultured bacterium**	0	0	0	0.469	0.183	0.257	0	0.303
**uncultured bacterium**	0.372	0.474	0.408	0.170	0.061	0.041	0.418	0.091
**uncultured bacterium**	0.421	0.398	0.436	0.172	0.081	0.041	0.418	0.098
**uncultured bacterium**	0.428	0.328	0.443	0.283	0.134	0.079	0.400	0.165
**uncultured bacterium**	0.424	0.360	0.531	0.124	0.066	0.073	0.438	0.088
**uncultured bacterium**	0	0	0	0.186	0.178	0.334	0	0.232
**Lactobacillus reuteri**	0.392	0.178	0.014	0.171	0.072	0.158	0.195	0.134
**Ambiguous_taxa**	0	0	0	0.054	0.163	0.150	0	0.122
**Lachnospiraceae bacterium A2**	0.328	0.354	0.472	0	0.010	0	0.385	0.003
**Ambiguous_taxa**	0.222	0.414	0.341	0.085	0.069	0.042	0.326	0.066
**uncultured bacterium**	0.005	0	0	0.255	0.347	0.433	0.002	0.345
**uncultured bacterium**	0.118	0.157	0.220	0.083	0.058	0.059	0.165	0.066
**Burkholderiales bacterium YL45**	0	0	0	0.409	0.497	1.083	0	0.663
**uncultured organism**	0.328	0	0.023	0	0	0	0.117	0
**mouse gut metagenome**	0.453	0.196	0.478	0.005	0	0	0.375	0.002
**Ambiguous_taxa**	0	0	0	0.623	0.283	0.168	0	0.358
**unidentified**	0.340	0.232	0.272	0.060	0.030	0.032	0.281	0.041
**Bacteroides thetaiotaomicron**	0	0	0	0.188	0.124	0.048	0	0.120
**Ambiguous_taxa**	0	0	0	0.110	0.219	0.159	0	0.163
**Clostridium sp. Culture-27**	0.120	0.323	0.180	0.006	0.014	0.015	0.208	0.012
**uncultured bacterium**	0.012	0.036	0.245	0.245	0.202	0.083	0.098	0.177
**Ambiguous_taxa**	0.291	0.137	0.190	0.006	0	0.003	0.206	0.003
**uncultured bacterium**	0	0	0	0.133	0.063	0.102	0	0.100
**Azospirillum sp. 47_25**	0	0	0	0.268	0.184	0.286	0	0.246
**uncultured bacterium**	0.057	0	0	0.156	0.282	0.201	0.019	0.213
**gut metagenome**	0	0	0	0.294	0.101	0.329	0	0.241
**uncultured Ruminococcaceae bacterium**	0	0.307	0	0.056	0.025	0.047	0.102	0.043
**D_6__uncultured bacterium**	0.267	0.164	0.259	0.084	0.026	0.019	0.230	0.043
**D_6__uncultured bacterium**	0	0	0	0	0.351	0	0	0.117
**D_6__uncultured bacterium**	0	0	0	0.272	0.058	0	0	0.110
**D_6__unidentified rumen bacterium JW32**	0	0	0	0.110	0.008	0.030	0	0.049
**D_6__Bacteroides massiliensis B84634**	0	0	0	0.407	0.350	0.074	0	0.277
**Ambiguous_taxa**	0	0	0	0.273	0.133	0	0	0.135
**uncultured bacterium**	0	0	0	0.151	0.064	0.145	0	0.120
**D_6__uncultured bacterium**	0	0	0	0.421	0.214	0.097	0	0.244
**D_6__uncultured bacterium**	0.012	0.023	0.138	0	0	0	0.058	0
**D_6__uncultured bacterium**	0	0	0	0.061	0.382	0.314	0	0.252
**Ambiguous_taxa**	0	0	0	0.015	0.193	0.054	0	0.087
**uncultured Barnesiella sp.**	0	0	0	0.008	0.172	0	0	0.060
**uncultured Clostridiales bacterium**	0	0	0	0.121	0.149	0.066	0	0.112
**Ambiguous_taxa**	0	0	0	0	0	0.157	0	0.052
**Ambiguous_taxa**	0	0	0	0	0.108	0	0	0.036
**Other < 0.5%**	1.806	1.665	1.655	3.319	2.603	1.817	1.709	2.579

Table S8. The α-diversity indexes for each sample and group

Sample	Observed	ACE	Shannon	Simpson	InvSimpson	Fisher
**0m SL 01**	199	199.93	4.185	0.967	30.162	27.524
**0m SL 02**	265	268.24	4.644	0.983	58.744	37.881
**0m SL 03**	186	186	4.139	0.969	32.010	25.309
**0m SL 04**	189	189	3.995	0.959	24.512	26.033
**0m SL 05**	213	213.30	4.292	0.972	35.445	29.211
**0m SL 06**	222	222.48	4.534	0.980	50.121	31.004
**0m SL 07**	224	224.25	3.864	0.944	17.856	30.198
**0m ML 01**	177	177.21	3.852	0.956	22.678	23.566
**0m ML 02**	190	190.22	3.841	0.953	21.176	25.559
**0m ML 03**	193	193	4.101	0.966	29.602	26.726
**0m ML 04**	245	245	4.539	0.977	42.600	34.122
**0m ML 05**	201	201	4.185	0.973	36.417	28.319
**0m ML 06**	201	201	4.027	0.960	25.025	27.584
**0m ML 07**	174	174.81	3.870	0.958	24.063	24.302
**0m WL 01**	208	208	4.022	0.958	23.627	28.251
**0m WL 02**	218	218.69	4.284	0.976	41.148	30.492
**0m WL 03**	194	194.64	4.026	0.966	29.335	26.567
**0m WL 04**	217	217.43	4.158	0.967	29.918	29.259
**0m WL 05**	217	217	4.438	0.978	44.447	29.980
**0m WL 06**	236	236.59	4.519	0.979	48.270	33.165
**0m WL 07**	204	204.15	3.986	0.961	25.935	27.525
**3m SL 01**	391	396.47	4.743	0.976	42.225	59.533
**3m SL 02**	402	402.56	4.995	0.987	79.858	62.239
**3m SL 03**	336	337.45	3.977	0.938	16.096	49.037
**3m SL 04**	303	303.81	4.458	0.976	41.912	45.009
**3m SL 05**	388	388.16	4.697	0.975	39.812	58.824
**3m SL 06**	334	334	4.484	0.968	31.535	52.379
**3m SL 07**	291	291.39	4.354	0.964	27.959	43.983
**3m ML 01**	292	292.94	3.759	0.905	10.545	42.807
**3m ML 02**	344	344.15	4.737	0.982	55.584	53.139
**3m ML 03**	377	377.16	4.689	0.977	44.180	56.914
**3m ML 04**	320	320.16	4.140	0.933	14.866	46.792
**3m ML 05**	318	319.11	3.969	0.946	18.426	47.032
**3m ML 06**	371	371.48	4.684	0.976	41.949	57.048
**3m ML 07**	292	292.37	4.284	0.967	30.047	42.589
**3m WL 01**	246	249.46	4.282	0.970	33.595	38.528
**3m WL 02**	249	249.36	4.350	0.977	43.466	36.444
**3m WL 03**	343	344.93	3.584	0.882	8.489	49.628
**3m WL 04**	291	291.31	4.344	0.969	32.714	43.496
**3m WL 05**	339	339.73	4.761	0.981	52.983	52.194
**3m WL 06**	295	295.16	3.878	0.911	11.210	42.963
**3m WL 07**	270	270.76	4.116	0.935	15.275	43.194

Group	Observed	ACE	Shannon	Simpson	InvSimpson	Fisher
**0m SL**	214	214.74	4.236	0.968	35.550	29.594
**0m ML**	197	197.46	4.059	0.963	28.794	27.168
**0m WL**	213	213.78	4.205	0.969	34.669	29.320
**3m SL**	349	350.55	4.530	0.969	39.914	53.000
**3m ML**	331	331.05	4.323	0.955	30.799	49.474
**3m WL**	290	291.53	4.188	0.946	28.248	43.778

Table S9. Co-occurrence network indices

Network indexes indices	0m SL	0m ML	0m WL	3m SL	3m ML	3m WL
**Node**	80	105	122	183	197	154
**Edge**	74	95	112	175	166	146
**Network Density**	0.023	0.017	0.02	0.011	0.009	0.012
**Assortativity**	-0.021	-0.013	-0.012	-0.008	-0.007	-0.008
**Module**	27	27	38	56	65	45
**Modularity (lc)**	0.778	0.881	0.873	0.960	0.969	0.956
